# Prognosis and long-term neurodevelopmental outcome in conservatively treated twin-to-twin transfusion syndrome

**DOI:** 10.1186/1471-2393-11-32

**Published:** 2011-04-22

**Authors:** Xiangqun Li, Seiichi Morokuma, Kotaro Fukushima, Yuka Otera, Yasuo Yumoto, Kiyomi Tsukimori, Masayuki Ochiai, Toshiro Hara, Norio Wake

**Affiliations:** 1 Department of Obstetrics and Gynecology, Graduate School of Medical Sciences, Kyushu University, Fukuoka, Japan; 2 Department of Obstetrics and Gynecology, Kyushu University Hospital, Kyushu University, Fukuoka, Japan; 3 Department of Pediatrics, Kyushu University Hospital, Kyushu University, Fukuoka, Japan; 4 Fukuoka Children's Hospital, Fukuoka, Japan

**Keywords:** fetus, TTTS, long-term outcome

## Abstract

**Background:**

Amnioreduction remains a treatment option for pregnancies with twin-to-twin transfusion syndrome (TTTS) not meeting criteria for laser surgery or those in which it is not feasible. Amnioreduction is a relatively simple treatment which does not require sophisticated technical equipment. Previous reports of conservative management have indicated that major neurodevelopmental impairment occurs in 14.3-26% of survivors. The purpose of this study was to investigate long-term neurodevelopmental outcome in conservatively treated TTTS.

**Methods:**

During the nine-year study period from January 1996 to December 2004, all pregnancies with TTTS who were admitted to our center were investigated. TTTS was diagnosed by using standard prenatal ultrasound criteria, and staged according to the criteria of Quintero *et al*. We reviewed gestational age at diagnosis, gestational age at delivery, the stage of TTTS at diagnosis, and diagnosis to delivery interval. Neonatal cranial ultrasound findings were reviewed and the neurodevelopmental outcomes were evaluated.

**Results:**

Twenty-one pregnancies with TTTS were included. Thirteen pregnancies (62%) were treated with serial amnioreduction. The mean gestational age at delivery was 28 weeks (22 - 34 weeks). The perinatal mortality rate was 42.9%. Twenty survivors were followed up until at least 3 years of age. The mean age at follow-up was 6.3 years (3 - 12 years). Six children (30%) had neurodevelopmental impairment. Four children (20%) had major neurodevelopmental impairment and two children (10%) had minor neurodevelopmental impairment. Children with neurodevelopmental impairment were delivered before 29 weeks of gestation.

**Conclusions:**

Our study showed a high rate of perinatal mortality and a high rate of major neurodevelopmental impairment in conservatively treated TTTS. The long-term outcomes for the survivors with TTTS were good when survivors were delivered after 29 weeks of gestation.

## Background

Twin-to-twin transfusion syndrome (TTTS) complicates 9% of monochorionic twin pregnancies, and if untreated, is associated with a perinatal loss rate of over 80% [1,2]. Fetal interventions, such as repeated serial amnioreduction and laser surgery may reduce the perinatal mortality rate. A recent meta analysis has shown that the overall survival rate varies from 57% to 77% following laser surgery and from 38% to 81% following serial amnioreduction [3].

The first randomized trial comparing amnioreduction with endoscopic laser surgery showed that endoscopic laser surgery resulted in higher survival rates and lower rates of neurologic complications at six months of age than did serial amnioreduction in severe TTTS presenting before 26 weeks of gestation. Therefore, endoscopic laser surgery has been adopted as the first-line treatment for TTTS diagnosed before 26 weeks [4]. As this technique interrupts placental vascular communication, it can potentially reduce neurodevelopmental impairment. Several studies, however, have reported that the incidence of neurodevelopmental impairment in TTTS survivors treated with laser surgery is still high [5,6].

Although amnioreduction is no longer the only treatment option for TTTS, a subset of patients may still benefit from this intervention. Amnioreduction is useful in the setting of TTTS not meeting criteria for laser surgery and in patients in whom laser surgery is not technically possible. Amnioreduction is a relatively simple treatment which does not require sophisticated technical equipment. Previous reports of conservative management have indicated that major neurodevelopmental impairment occurs in 14.3%- 26% of survivors [7-10].

This study was undertaken to investigate the long-term neurodevelopmental outcome in conservatively treated TTTS.

## Methods

We examined the prenatal records of all women and the medical records of children and neonates with TTTS admitted to the Maternity and Perinatal Care Unit of Kyushu University Hospital from January 1996 to December 2004. TTTS was diagnosed in all cases by ultrasound criteria of polyhydramnios (> 8 cm, deepest vertical pool) in one twin sac and oligohydramnios (‹2 cm, deepest vertical pool) in the co-twin of a monochorionic, diamniotic pregnancy [11]. In our hospital, laser surgery was not performed, so cases with laser surgery were not included.

The following data were obtained from the maternal medical record: gestational age at diagnosis, gestational age at delivery, the stage of TTTS at diagnosis, diagnosis to delivery interval, and amnioreduction. TTTS was staged according to the criteria of Quintero et al. [12]. The following neonatal data were extracted: birth weight and cranial ultrasound findings. Cranial ultrasound scans were obtained in all neonates shortly after birth and further scans were carried out one, three, five, and seven days after birth, followed by weekly scans thereafter until discharge. Additional scanning was undertaken as clinically indicated. Abnormal cranial ultrasound findings in the neonatal period were reviewed for the presence of intraventricular hemorrhage (IVH), periventricular leukomalacia (PVL), and ventricular dilatation. Intraventricular hemorrhage (IVH) was classified according to Papile et al. [13]. Periventricular leukomalacia (PVL) was graded according to Vries et al. [14]. Severe cerebral lesions on cranial ultrasound findings were defined as the presence of IVH grade III or IV, and/or PVL ≥ grade II.

All surviving children were offered care in our high-risk infant follow-up program. Repetitive examinations by experienced pediatric neurologists were performed. Their development was evaluated using the Enjoji Developmental Scale [15] before six years of age, followed by the Japanese Wechsler Intelligence Scale for Children-Revised [16] (WISC-R) or Wechsler Preschool Primary Scale of Intelligence at six years or older [17] (WPPSI). Children with normal development had a normal neurological evaluation and developmental quotient (DQ) or intellectual quotient (IQ) > 85. Minor neurodevelopmental impairment was defined as the presence of at least one of the following: mild cerebral palsy causing motor clumsiness or non-fluent gait, DQ or IQ between 70 and 85. Major neurodevelopmental impairment was defined as the presence of at least one of the following: cerebral palsy, DQ or IQ < 70, deafness, or blindness. Cerebral palsy was classified as diplegia, hemiplegia, quadriplegia, dyskinetic or mixed [6,8]. Developmental or mental borderline was defined as DQ or IQ between 70 and 85. Developmental or mental retardation was defined as DQ or IQ < 70 [15-17].

### Statistical analysis

Analysis was performed with the SPSS statistical package 16.0 (SPSS Inc, Chicago, IL). Differences between categorical variables were analyzed using the chi-square test or the Fisher exact test as appropriate. Differences between continuous variables were tested using independent sample t-tests. A statistically significant difference was defined as p < 0.05.

### Ethical considerations

Ethical permission was obtained, based on the criteria of the Kyushu University research ethics committee concerning observational studies.

## Results

Table 1 shows the pregnancy and fetal outcomes with TTTS. During the nine-year study, 21 pregnancies with TTTS were admitted to our center. The gestational age at diagnosis ranged from 18 to 34 weeks with a mean age of 24 weeks. The gestational age at delivery ranged from 22 to 34 weeks with a mean age of 28 weeks. Thirteen pregnancies (61.9%) were treated with serial amnioreduction, with the number of amniocenteses ranging from one to six per pregnancy (mean 2.7). In the eight pregnancies in which amnioreduction was not performed, one pregnancy was complicated by premature rupture of the membrane, two pregnancies presented with mild TTTS (stage I), and one pregnancy opted for delivery. There were two survivors in 42.9% of pregnancies, one survivor in 19% of pregnancies, and no survivors in 38.1% of the pregnancies. The Quintero stage at diagnosis showed 11 pregnancies to be stage I, four pregnancies to be stage II, three pregnancies to be stage III, three pregnancies to be stage IV, and none to be stage V. Intrauterine fetal demise occurred in five fetuses (11.9%), and all of these resulted from pregnancies not treated with amnioreduction. One pregnancy culminated in the intrauterine death of both fetuses, and three pregnancies resulted in the intrauterine death of one fetus. In the three neonates born after the intrauterine fetal demise of their co-twin, one neonate born at 22.4 weeks died soon after birth, one neonate died of heart failure at 75 days of age, and one survivor had mild cerebral palsy with epilepsy and mental retardation. Neonatal death occurred in 13 neonates (35.1%). The overall perinatal mortality rate was 42.9% (18/42). Two infants died after the neonatal period. Twenty-two survivors were followed to at least the age of 1 year corrected for prematurity. Two survivors were lost to follow-up after the age of one year. Neurological follow-up was available for the remaining twenty survivors. The mean age at follow-up was 6.3 years (range, 3-12 years). Fourteen children (70%) had normal neurological development. Six children (30%) had neurodevelopmental impairment.

**Table 1:** Antenatal and delivery characteristics

Pregnancies(n)	21
Age (years)	28 (20-38)
Parity	0 (0-3)
Gestational age at diagnosis(weeks)	24 (18-34)
Gestational age at delivery(weeks)	28 (22-34)
Diagnosis to delivery interval(days)	25 (1-73)
Use of amnioreduction	13 (61.9%)
Amnioreduction per pregnancy	2.7 (1-6)
Cesarean section	13 (61.9%)
Gestational age < 30 weeks at delivery	15 (71.4%)
Quintero stage I	11 (52.4%)
Quintero stage II	4 (19%)
Quintero stage III	3 (14.3%)
Quintero stage IV	3 (14.3%)
Quintero stage V	0
IUFD of 1 twin	3 (14.3%)
IUFD of both twins	1 (4.8%)
0 survivor*	8 (38.1%)
1 survivor	4 (19%)
2 survivors	9 (42.9%)
	
Fetus(n)	42
Intrauterine fetal death (IUFD)	5 (11.9%)
Neonatal death	13 (35.1%)
Overall perinatal mortality	18 (42.9%)
Infant death	2 (5.4%)
Lost to follow-up	2 (5.4%)
Long-term survivors	20 (54.1%)
neurodevelopmental impairment	6 (30%)

Data shown as mean (range) or n (%) as appropriate.

*Survivor is defined as the survival of at least 12 months of age.

Of 37 neonates, four died before the cranial ultrasound scan was performed. Abnormal ultrasound findings were present in seventeen (51.5%) of 33 neonates as follows: IVH grade I in five neonates; IVH grade І in three neonates; IVH grade I + III in two neonates; PVL grade I in three neonates; PVL grade І in two neonates; and mild ventricular dilatation in two neonates. Of seventeen neonates with abnormal cranial ultrasound findings, neonatal death occurred in five neonates; infant death occurred in two infants; one survivor was lost to follow-up; five of the nine survivors had neurodevelopmental impairment. A severe cerebral lesion was present in four neonates (12.1%). One neonate born at 24 weeks died of heart failure at one day of age. One neonate whose co-twin died at 26 weeks of gestation died of heart failure at 75 days of age. Two survivors had major neurodevelopmental impairment. On 16 neonates with normal cranial ultrasound findings, neonatal death occurred in four neonates; one survivor was lost to follow-up; and one survivor had minor neurodevelopmental impairment.

Table 2 shows adverse neurodevelopmental outcomes with TTTS. Four children (20%) had major neurodevelopmental impairment. For case 1 (in which fetal death of the donor co-twin occurred at 25 weeks of gestation), birth occurred at 27 weeks of gestation. The child was found to have mental retardation (IQ 61) and epilepsy with mild cerebral palsy causing a non-fluent gait. Case 2 (PVL (grade І)) had mental retardation (IQ 69). Case 3 had mild bilateral ventricular dilatation and mental retardation (IQ 54). Case 4 had posthemorrhagic hydrocephalus and mild cerebral palsy causing motor clumsiness. Because the child's IQ was exactly 70, the case was classified as borderline mental impairment. However, we considered case 4 to have major neurodevelopmental impairment in this study on the basis of general clinical judgment.

**Table 2:** Data of the 6 surviving twins with adverse neurodevelopmental outcomes

Case	D/R	Quintero stage	GA at diagnosis (weeks)	GA at birth (weeks)	AR (n)	Birth weight (g)	Neonatal cranial ultrasound findings	Follow-up age (years)	Neurodevelopmental Impairment	IQ	Outcome of co-twin	Severity
1	R	III	19	27	0	814	IVH I	6	Mild CP, epilepsy, Mental retardation	61(6 years)	IUFD	major
2	R	I	20	28	4	1018	PVL І	8	Mental retardation	69(6 years)	mental borderline	major
3	R	II	26	27	1	1130	mild bilateral ventricular dilatation	7	Mental retardation	54(6 years)	Infant death	major
4	D	I	21	28	0	865	IVH III	12	Mild CP, mental borderline hydrocephalus	70(12 years)	Mild CP, mental borderline	major
5	R	I	21	28	0	975	IVH I	12	Mild CP, Mental borderline	78(12 years)	Mild CP, mental borderline, hydrocephalus	minor
6	D	I	20	28	4	886	normal	8	Mental borderline	71(6 years)	mental retardation	minor

R = Recipient, D = Donor, GA = Gestational age, AR = Amnioreductions

IVH = Intraventricular hemorrhage, PVL = periventricular leukomalacia; CP = cerebral palsy,

IUFD = intrauterine fetal death, IQ = intellectual quotient.

Two children (10%) had minor neurodevelopmental impairment. Case 5 had borderline mental impairment (IQ 78) and mild cerebral palsy causing motor clumsiness while case 6 had borderline mental impairment only (IQ 71). Case 2/case 6 and case 4/case 5 comprised the pairs in two twin pregnancies. All of them were delivered before 29 weeks of gestation.

Table 3 shows antenatal and delivery characteristics in surviving cases without neurodevelopmental complications as well as those complicated by death or neurodevelopmental impairment. The mean gestational age for those surviving without complication at diagnosis and delivery was 28 weeks (range, 24 - 34 weeks) and 31 weeks (range, 27-34 weeks), respectively and the mean gestational age for those with death or neurodevelopmental impairment at diagnosis and delivery was 22 weeks (range, 19 - 27 weeks) and 27 weeks (range, 22 - 31 weeks), respectively. Overall, the mean gestational age for those with death or neurodevelopmental impairment was lower than surviving cases without neurodevelopmental impairment. The interval between diagnosis and delivery was significantly longer and the birth weight was significantly lower in those complicated by death or neurodevelopmental impairment. There was no difference in the ratio of Donors to Recipients between the groups.

**Table 3:** Comparison of normal development and death or neurodevelopmental impairment

	Normal development	Death or Neurodevelopmental impairment	*P *value
Number	14	26	
Gestational age at diagnosis (weeks)	28 (24-34)	22 (19-27)	< 0.001
Gestational age at delivery (weeks)	31 (27-34)	27 (22-31)	< 0.001
Diagnosis to delivery interval (days)	15 (2-34)	32 (1-50)	0.002
Birth weight (g)	1355 (750-2130)	812 (255-1970)	0.001
Donor/Recipient	7/7	13/13	0.999

Data shown as mean (range) or n as appropriate.

Table 4 shows perinatal outcomes and survivors' outcomes between amnioreduction and non-amnioreduction. There was no significant difference in the mean gestational age at delivery, diagnosis to delivery interval, or neurodevelopmental impairment.

**Table 4:** Perinatal outcomes and survivor' outcomes between amnioreduction (AR) and non-amnioreduction (non-AR)

	AR	non-AR	*P *value
Number	26	16	*NS*
Gestational age at diagnosis (weeks)	25 (18-32)	23 (19-34)	*NS*
Gestational age at delivery (weeks)	28 (24-34)	27 (22-34)	*NS*
Diagnosis to delivery interval (days)	22 (4-56)	30 (1-73)	*NS*
Birth weight (g)	1124 (335-2222)	921 (255-2138)	*NS*
IUFD	0	5 (31.2%)	*NS*
Neonatal death	10 (38.5%)	3 (27.3%)	*NS*
Infant death	1 (3.8%)	1 (9.1%)	*NS*
Lost to follow-up	2 (7.7%)	0	*NS*
Long-term survivors	13 (50%)	7 (63.6%)	*NS*
Neurodevelopmental impairment	3 (23.1%)	3 (42.9%)	*NS*

Data shown as mean (range) or n (%) as appropriate.

NS, not significant; IUFD, intrauterine fetal death.

## Discussion

In this study, we reported that the overall perinatal mortality rate in conservatively treated patients with TTTS was 42.9%. The result was similar to the mortality reported by Mari *et al*. [18], Gray *et al*. [19], and Senat *et al*. [4].

We found that 30% of surviving twins had neurodevelopmental impairment when TTTS was treated conservatively. Four children (20%) had major neurodevelopmental impairment. Two children (10%) had minor neurodevelopmental impairment. Cincotta et al. observed 17 pregnancies, of which 12 (71%) were treated with serial amnioreduction [9]. Twenty-three children were followed up at least two years. Five survivors (22%) had major neurological morbidity, which was similar to our results. Dickinson et al. [7] investigated 52 surviving infants from 31 pregnancies, with 22 pregnancies treated with serial amnioreduction. 49 children were followed up at a median age of five years. Major neurodevelopmental disability was present in seven children (14.3%). Minor neurodevelopmental impairment was not reported. Mari et al. investigated 42 survivors of TTTS after aggressive amnioreduction [20]. Cerebral palsy was diagnosed in two of 42 infants (4.7%). No developmental tests were used and the incidence of major neurodevelopmental impairment was lower. With regard to overall neurodevelopmental impairment, we found the incidence was lower than several studies previously reported in analyses of long-term neurological outcome [8,10,21]. Lopriore et al. investigated 29 pregnancies, including 18 pregnancies (62%) with serial amnioreduction [10]. Twenty-nine children were followed up to a mean age of 6.2 years (4-11). Major neurodevelopmental impairment was observed in 21% of children, and minor neurodevelopmental impairment (mild speech delay) was seen in 17.2% (5/29). Haverkamp et al. described 40 children who were followed up to a mean age of 24 months [21]. Major neurodevelopmental impairment was seen in 23%, while minor neurodevelopmental impairment was found in 33%. Frusca et al. investigated 31 children who were followed up to a mean age of 24 months [8]. Eight children (25.8%) had major neurologic disabilities; five children (16.1%) had minor neurologic disabilities. This discrepancy may be due to the heterogeneity within the neurodevelopmental impairment of TTTS and assessment of neurodevelopmental outcome.

Abnormal cranial ultrasound findings were found in seventeen cases (51.5%) of the 33 neonates who underwent cranial ultrasound scans. Denbow *et al*. reported an even higher incidence (58%) of neonates with abnormal cranial ultrasound findings [2]. Gestational age at delivery and birth weight were not associated with an incidence of abnormal cranial ultrasound findings. Cranial ultrasound scans at birth are commonly used as a surrogate marker for neurodevelopmental outcome in later life [22]. Periventricular white matter lesions (WMLs) and persistent ventriculomegaly in particular have been associated with an adverse neurodevelopmental outcome [22,23]. In our patient group, five cases (15.2%) showed WMLs. Some studies have shown evidence of cerebral white matter lesions in one third of monochorionic twin infants at birth, particularly when the pregnancy was complicated by long-term coexistence with a co-twin intrauterine demise [24,25]. Hecher *et al*. [26] reported a lower incidence (6%) of cerebral WMLs following laser surgery, compared with the cases treated by amnioreduction (18%). Results from observational studies [2,10,27] and an international multicenter registry [18,28] of TTTS treated by amnioreduction suggest that the incidence of major cranial abnormalities ranges between 18 and 41%. Discrepancies among results may be due to differences in diagnostic criteria, disease onset, severity of the TTTS, treatment modalities, and stratification of cranial lesions.

Since laser surgery for TTTS was introduced, survival rates have been increasing. Neurodevelopmental impairment, however, is still relatively common. Sutcliffe et al. investigated 67 children with severe TTTS treated by laser surgery, and 9% had cerebral palsy [29]. They did not report the number of infants with developmental delay. Banek et al. investigated 89 children after TTTS treated by laser surgery between 14 months and 44 months. Eleven percent of the children had minor neurologic deficiencies, and an equal portion had major neurologic deficiencies [5]. Graef et al. investigated 167 children from TTTS treated by laser surgery and followed to a median age of 38 months (14 - 53) [30]. In this group, 7.2% of the children showed minor neurologic abnormalities, and 6% showed major neurologic abnormalities. Both studies originated from the same research group in Germany. The largest analysis concerning long-term neurodevelopmental outcome after TTTS with laser surgery was published by Lopriore et al. [6]. They investigated 278 children at two years of age (corrected for prematurity). The incidence of major neurodevelopmental impairment was 18%. They did not report minor neurodevelopmental impairment.

Whether TTTS is treated with laser surgery or managed conservatively, the incidence of major neurodevelopmental impairment is high. The pathogenesis of cerebral injury in TTTS is not clearly defined. Cerebral injury in TTTS may result from antenatal injury secondary to hemodynamic and hematological imbalance [21] and/or from postnatal injury associated with prematurity [31] and low birth weight [8]. In our study, the long-term outcomes for the survivors with TTTS were relatively good when survivors were delivered after 29 weeks of gestation. Mari et al. found that long-term outcomes for the twins with TTTS were excellent when both fetuses were delivered alive after 27 weeks of gestation [20]. A research group from Germany reported that neurodevelopmental disability in infants who were born before 32 weeks of gestation was significantly higher [5,30]. Lopriore et al. and Lenclen et al. showed that early gestational age at delivery was a risk factor for neurodevelopmental impairment [6,32]. It seems that prolongation of gestation is central in management strategies.

The results of the present study suggest that the outcomes, particularly neurodevelopmental, resulting from the conservative management of TTTS are not markedly different from outcomes obtained with laser surgery. The strength of our conclusion is limited by the small study size and the preponderance of mild cases which presented later[6]. While immediate neonatal survival may be improved with laser surgery, this advantage may be lost with long term follow-up.

## Conclusions

In conclusion, the primary predictor for neurodevelopmental impairment is gestational age, regardless of whether the management is conservative or by laser surgery. Therefore, prolongation of gestation is important. Additionally, we suggest performing a routine cranial ultrasound examination after birth as the results will be valuable in predicting long-term neurodevelopmental outcomes.

## Competing interests

The authors declare that they have no competing interests.

## Authors' contributions

XL, SM and KF examined and drafted the manuscript. YO, YY and MO TH, KT and NW participated in the design of the study and coordination. YO, YY and MO collected data and performed statistical examination. All authors read and approved the final manuscript.

## Pre-publication history

The pre-publication history for this paper can be accessed here:

http://www.biomedcentral.com/1471-2393/11/32/prepub

## References

[B1] Lewi L Jani J Blickstein I Huber A Gucciardo L Van Mieghem T Done E Boes AS Hecher K Gratacos E Lewi P Deprest J The outcome of monochorionic diamniotic twin gestations in the era of invasive fetal therapy: a prospective cohort study Am J Obstet Gynecol 2008 199 514e1 8 10.1016/j.ajog.2008.03.050 18533114

[B2] Denbow ML Battin MR Cowan F Azzopardi D Edwards AD Fisk NM Neonatal cranial ultrasonographic findings in preterm twins complicated by severe fetofetal transfusion syndrome Am J Obstet Gynecol 1998 178 479 83 10.1016/S0002-9378(98)70424-7 9539512

[B3] Rossi AC D'Addario V Laser therapy and serial amnioreduction as treatment for twin-twin transfusion syndrome: a metaanalysis and review of literature Am J Obstet Gynecol 2008 198 147 52 10.1016/j.ajog.2007.09.043 18068144

[B4] Senat MV Deprest J Boulvain M Paupe A Winer N Ville Y Endoscopic laser surgery versus serial amnioreduction for severe twin-to-twin transfusion syndrome N Engl J Med 2004 351 136 44 10.1056/NEJMoa032597 15238624

[B5] Banek CS Hecher K Hackeloer BJ Bartmann P long-term neuordevelopmental outcome after intrauterine laser treatment for severe twin-twin transfusion syndrome Am J Obstet Gynecol 2003 188 876 80 10.1067/mob.2003.202 12712079

[B6] Lopriore E Ortibus E Acosta-Rojas R Le Cessie S Middeldrop JM Oepkes D Gratacos E Vandenbussche FPHA Deprest J Walther FJ Lewi L Risk factors for neurodevelopment impairment in twin-twin transfusion syndrome treated with fetoscopic laser surgery Obstet Gynecol 2009 113 361 6 19155907 10.1097/AOG.0b013e318195873e

[B7] Dickinson JE Duncombe GJ Evans SF French NP Hagan R The long term neurologic outcome of children from pregnancies complicated by twin-to-twin transfusion syndrome BJOG 2005 112 63 8 10.1111/j.1471-0528.2004.00330.x 15663399

[B8] Frusca T Soregaroli M Fichera A Taddei F Villani P Accorsi P Martelli P Pregnancies complicated by twin-twin transfusion syndrome: outcome and long-term neurological follow-up Eur J Obstet Gynecol Reprod Biol 2003 107 145 50 10.1016/S0301-2115(02)00341-X 12648859

[B9] Cincotta RB Gray PH Phythian G Rogers YM Chan FY Long term outcome of twin-twin transfusion syndrome Arch Dis Child Fetal Neonatal Ed 2000 83 171 6 10.1136/fn.83.3.F171 PMC1721175 11040163

[B10] Lopriore E Nagel HT Vandenbussche FP Walther FJ Long-term neurodevelopmental outcome in twin-to-twin transfusion syndrome Am J Obstet Gynecol 2003 189 1314 9 10.1067/S0002-9378(03)00760-9 14634561

[B11] Wittmann BK Baldwin VJ Nichol B Antenatal diagnosis of twin transfusion syndrome by ultrasound Obstet Gynecol 1981 58 123 7 7017517

[B12] Quintero RA Morales WJ Allen MH Bornick PW Johnson PK Kruger M Staging of twin-twin transfusion syndrome J Perinatol 1999 19 550 5 10.1038/sj.jp.7200292 10645517

[B13] Papile LA Burstein J Burstein R Koffler H Incidence and evolution of subependymal and intraventricular hemorrhage: a study of infants with birth weights less than 1500 gm J Pediatr 1978 92 529 34 10.1016/S0022-3476(78)80282-0 305471

[B14] de Vries LS Eken P Dubowitz LM The spectrum of leukomalacia using cranial ultrasound Behav Brain Res 1992 49 1 6 10.1016/S0166-4328(05)80189-5 1388792

[B15] Enjoji M Yanai N Analytic test for development in infancy and childhood Paediatr Jpn 1961 4 2 6

[B16] Wechsler D Wechsler Intelligence Scale for Children, Revised 1974 New York, Psychological Corporation

[B17] Wechsler D Manual for the Wechsler Preschool and Primary Scale of Intelligence 1989 New York, Psychological corporation

[B18] Mari G Roberts A Detti L Kovanci E Stefos T Bahado-Singh RO Deter RL Fisk NM Perinatal morbidity and mortality rates in severe twin-twin transfusion syndrome: results of the international amnioreduction registry Am J Obstet Gynecol 2001 185 708 15 10.1067/mob.2001.117188 11568802

[B19] Gray PH Cincotta R Chan FY Soong B Perinatal outcomes with laser surgery for twin-twin transfusion syndrome Twin Res Hum Genet 2006 9 438 43 16790154 10.1375/183242706777591371

[B20] Mari G Detti L Oz U Abuhamad AZ Long-term outcome in twin-twin transfusion syndrome treated with serial aggressive amnioreduction Am J Obstet Gynecol 2000 183 211 7 10920333 10.1067/mob.2000.105583

[B21] Haverkamp F Lex C Hanisch C Fahnenstich H Zerres K Neurodevelopmental risks in twin-to-twin transfusion syndrome: preliminary findings Eur J Paediatr Neurol 2001 5 21 7 11277360 10.1053/ejpn.2001.0400

[B22] Whitaker AH Feldman JF Van Rossem R Schonfeld IS Pinto-Martin JA Torre C Blumenthal SR Paneth NS Neonatal cranial ultrasound abnormalities in low birth weight infants: relation to cognitive outcomes at six years of age Pediatrics 1996 98 719 29 8885952

[B23] Pinto-Martin JA Riolo S Cnaan A Holzman C Susser MW Paneth N Cranial ultrasound prediction of disabling and nondisabling cerebral palsy at age two in a low birth weight population Pediatrics 1995 95 249 54 7838643

[B24] Grafe MR Antenatal cerebral necrosis in monochorionic twins Pediatr Pathol 1993 13 15 9 10.3109/15513819309048188 8474948

[B25] Bejar R Vigliocco G Gramajo H Solana C Benirschke K Berry C Coen R Resnik R Antenatal origin of neurologic damage in newborn infants. II. Multiple gestations Am J Obstet Gynecol 1990 162 1230 6 2187353 10.1016/0002-9378(90)90024-2

[B26] Hecher K Plath H Bregenzer T Hansmann M Hackeloer BJ Endoscopic laser surgery versus serial amniocenteses in the treatment of severe twin-twin transfusion syndrome Am J Obstet Gynecol 1999 180 717 24 10.1016/S0002-9378(99)70278-4 10076153

[B27] Hikino S Ohga S Kanda T Nakashima T Yamamoto J Nakayama H Nakano H Hara T Long-term outcome of infants with twin-to-twin transfusion syndrome Fetal Diagn Ther 2007 22 68 74 10.1159/000095847 17003558

[B28] Dickinson JE Evans SF Obstetric and perinatal outcomes from the Australian and New Zealand twin-twin transfusion syndrome registry Am J Obstet Gynecol 2000 182 706 12 10.1067/mob.2000.104236 10739534

[B29] Sutcliffe AG Sebire NJ Pigott AJ Taylor B Edwards PR Nicolaides KH Outcome for children born after in utero laser ablation therapy for severe twin-to-twin transfusion syndrome BJOG 2001 108 1246 50 11843386 10.1111/j.1471-0528.2001.00294.x

[B30] Graef C Ellenrieder B Hecher K Hacheloer BJ Huber A Bartmann P Long-term neurodevelopmental outcome of 167 children after intrauterine laser treatment for severe twin-twin transfusion syndrome Am J Obstet Gynecol 2006 194 303 8 10.1016/j.ajog.2005.07.040 16458621

[B31] Lutfi S Allen VM Fahey J O'Connell CM Vincer MJ Twin-twin transfusion syndrome: a population-based study Obstet Gynecol 2004 104 1289 97 10.1097/01.AOG.0000143828.41271.6c 15572492

[B32] Lenclen R Ciarlo G Paupe A Bussieres L Ville Y Neurodevelopmental outcome at 2 years in children born preterm treated by amnioreduction or fetoscopic laser surgery for twin-to-twin transfusion syndrome: comparison with dichorionic twins Am J Obstet Gynecol 2009 201 291.e1 5 10.1016/j.ajog.2009.05.036 19608152

